# Comparative Molecular Profiling of the PPARα/γ Activator Aleglitazar: PPAR Selectivity, Activity and Interaction with Cofactors

**DOI:** 10.1002/cmdc.201100598

**Published:** 2012-04-04

**Authors:** Michel Dietz, Peter Mohr, Bernd Kuhn, Hans Peter Maerki, Peter Hartman, Armin Ruf, Jörg Benz, Uwe Grether, Matthew B Wright

**Affiliations:** a Pharma Research & Early Development (pRED), F. Hoffmann-La Roche AGGrenzacherstrasse 124, Basel 4070 (Switzerland); b Discovery TechnologiesF. Hoffmann-La Roche AG, Grenzacherstrasse 124, Basel 4070 (Switzerland); c Discovery ChemistryF. Hoffmann-La Roche AG, Grenzacherstrasse 124, Basel 4070 (Switzerland); d DTA CVM, F. Hoffmann-La Roche AGGrenzacherstrasse 124, Basel 4070 (Switzerland)

**Keywords:** aleglitazar, cofactors, nuclear receptors, peroxisome proliferator-activated receptors (PPARs), transcription

## Abstract

Peroxisome proliferator-activated receptors (PPARs) are a family of nuclear hormone receptors that control the expression of genes involved in a variety of physiologic processes, through heterodimerization with retinoid X receptor and complex formation with various cofactors. Drugs or treatment regimens that combine the beneficial effects of PPARα and γ agonism present an attractive therapeutic strategy to reduce cardiovascular risk factors. Aleglitazar is a dual PPARα/γ agonist currently in phase III clinical development for the treatment of patients with type 2 diabetes mellitus who recently experienced an acute coronary event. The potency and efficacy of aleglitazar was evaluated in a head-to-head comparison with other PPARα, γ and δ ligands. A comprehensive, 12-concentration dose–response analysis using a cell-based assay showed aleglitazar to be highly potent, with EC_50_ values of 5 nm and 9 nm for PPARα and PPARγ, respectively. Cofactor recruitment profiles confirmed that aleglitazar is a potent and balanced activator of PPARα and γ. The efficacy and potency of aleglitazar are discussed in relation to other dual PPARα/γ agonists, in context with the published X-ray crystal structures of both PPARα and γ.

## Introduction

Peroxisome proliferator-activated receptors (PPARs) belong to a family of ligand-regulated nuclear hormone receptors that control the expression of genes involved in a variety of physiologic processes, including lipid and glucose homeostasis, inflammation, and cell differentiation.^1^, ^2^ The fibrates, which act as PPARα agonists, are used clinically to treat dyslipidemia and associated cardiovascular risk.^3^, ^4^ Thiazolidinedione (TZD) PPARγ agonists, such as pioglitazone and rosiglitazone, improve insulin sensitivity and glucose homeostasis,^5^ and exhibit anti-inflammatory^6^ and antihypertensive effects.^7^–^9^ However, the use of TZDs is associated with weight gain, increased incidence of edema, and risk of congestive heart failure.^10^, ^11^ Illustrating the distinct compound-specific effects of TZDs, pioglitazone has been shown to reduce atheroma in patients with type 2 diabetes mellitus^12^, ^13^ and to decrease cardiovascular events in some studies,^14^ whereas rosiglitazone increases the risk of myocardial infarction.^15^ Clinical trials and post-marketing surveys support the notion that rosiglitazone and pioglitazone do not share the hepatotoxic profile of the prototype TZD PPARγ agonist troglitazone,^16^ further highlighting the distinct compound-specific effects of TZDs.

Drugs or treatment regimens that combine the beneficial effects of PPARα and γ agonism present an attractive therapeutic strategy.^17^, ^18^ Several dual PPARα/γ agonists, namely muraglitazar, tesaglitazar and aleglitazar,^19^ have reached late-stage clinical trials. The development of muraglitazar and tesaglitazar was discontinued due to compound-specific side effects that included elevated risk of cardiovascular events for muraglitazar^20^ and decreased renal function for tesaglitazar.^21^ Moreover, in clinical studies, both muraglitazar and tesaglitazar increased weight gain and edema to a similar or even greater degree than pioglitazone.^22^, ^23^ In contrast, in the phase II SYNCHRONY trial (NCT00388518) in patients with type 2 diabetes mellitus, aleglitazar caused less weight gain and demonstrated better lipid effects than pioglitazone at doses achieving similar glycemic control, although the study was not designed to assess significant differences between the two treatments.^24^

The regulation of PPAR activity is complex. PPARs are regulated through mechanisms including phosphorylation and dephosphorylation,^25^, ^26^ ligand- and cell-specific interactions with cofactors of the p160 family,^27^ and heterodimerization with members of the retinoid X receptor (RXR) family.^27^ The specific cofactors recruited to PPAR–RXR complexes in response to different ligands are suggested to lead to major differences in transactivation of target genes.^27^–^29^ Many of these cofactors have been shown in their own right to be key players in metabolic regulation.^30^, ^31^ It has thus been hypothesized that the balance between efficacy and side-effect profiles of each specific PPAR agonist might relate, at least in part, to its potency, PPAR isoform selectivity, and/or pattern of cofactor recruitment. New molecules designed taking these factors into account have the potential to become superior therapeutics that sufficiently separate efficacy from side effects, leading to a broader therapeutic window. This concept has led to efforts to identify selective PPAR modulators, such as the partial PPARγ agonists INT131,^32^ MK0533,^33^ and ATx008-001/FK614.^33^ INT131 recruits DRIP205, a co-activator involved in adipocyte differentiation, with an efficacy of about 20–25 % that of prototypical full PPARγ agonists, including rosiglitazone and pioglitazone.^32^ In animal models of diabetes, INT131 caused less weight gain compared with pioglitazone or rosiglitazone, while retaining efficacy to reduce plasma glucose.^32^, ^34^ The aim of dual PPARα/γ agonist treatment is to simultaneously capture the glycemic benefits of targeting PPARγ and the lipid benefits of targeting PPARα. Indeed, there is evidence indicating distinct but overlapping gene signature profiles for different PPAR agonists.^35^–^37^ In this context, aleglitazar, a dual PPARα/γ agonist currently in phase III development, has been shown to induce transcriptional signatures different from those of other dual PPARα/γ treatments.^35^ This could underlie the favorable efficacy/side-effect profile observed in preclinical and clinical investigations.^24^, ^38^

**Figure 1:**
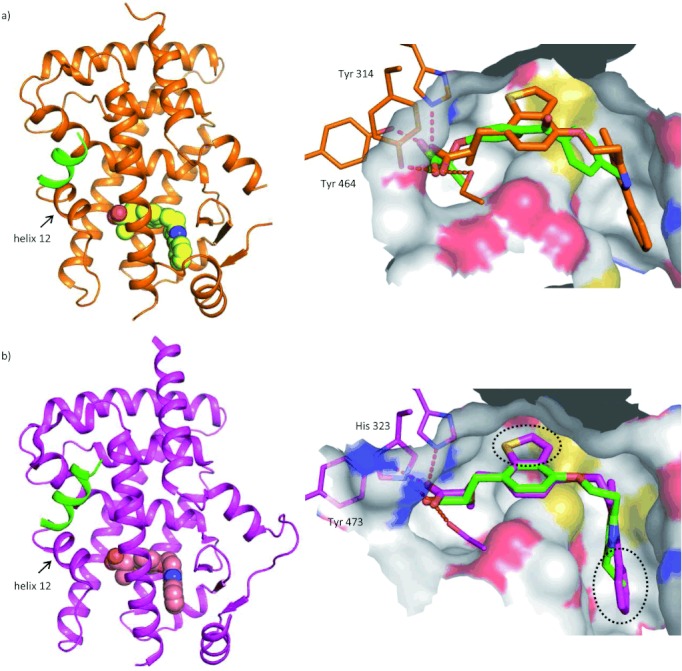
Ribbon diagram and close-up view of the ligand binding pockets derived from co-crystal structures of aleglitazar in a ternary complex with a) the PPARα ligand binding domain and a 13-residue fragment of SRC1 motif 3 (KDHQLLRYLLDKD) (PDB: 3G8I),^19^ and b) the PPARγ ligand binding domain and a 13-residue fragment of SRC1 motif 1 (QTSHKLVQLLTTT) (PDB: 3G9E).^19^ The SRC1 fragments are shown in green and aleglitazar is shown in space-filling model. Protein–ligand hydrogen bonds are shown as red, dashed lines. For comparison, models of fenofibrate in PPARα (based on the X-ray complex with GW735, PDB: 2P54)^53^ and pioglitazone in PPARγ (based on the X-ray complex with rosiglitazone, PDB: 1FM6)^54^ are overlaid onto aleglitazar in the right panels. The dotted ellipses show additional hydrophobic interaction atoms present in aleglitazar that are not present in pioglitazone.

We recently solved the X-ray structures of the ternary complexes of aleglitazar with a peptide fragment of the receptor co-activator SRC1 and the ligand binding domains of both PPARα and γ.^19^ As shown in the structural representations in Figure 1, the C-terminal activation helix 12 in both PPARs adopts a full-agonist conformation, mediated by a direct interaction of the carboxylate head group of aleglitazar with Tyr 464 and Tyr 473 of the α- and γ-isoforms, respectively. This arrangement of helix 12 generates a hydrophobic interaction surface to which the LxxLL motif of the co-activator SRC1 fragment peptide binds. The carboxylate of aleglitazar engages in three additional strong hydrogen-bonding interactions with Ser 280, Tyr 314 and His 440 residues of PPARα or Ser 289, His 323 and His 449 residues of PPARγ. Moreover, the extended aleglitazar structure has excellent shape complementarity with both the PPARα and PPARγ ligand binding pockets. The central benzothiophene and terminal phenyl ring of aleglitazar make additional hydrophobic interactions that contribute to greater binding efficiency in both receptors compared with other ligands.

Here, we describe the relative potency and efficacy of aleglitazar in a head-to-head comparison with key PPAR ligands, including dual PPARα/γ agonists previously in development, as well as marketed PPAR drugs (Table 1). Comprehensive transcriptional transactivation and cofactor recruitment studies confirm the high potency and balanced activity of aleglitazar on PPARα and γ and suggest, particularly for PPARα, that aleglitazar possesses a unique profile compared with other ligands.

**Table 1:** The structure and PPAR selectivity of ligands used in this study.

Compd	Structure	Selectivity
Aleglitazar	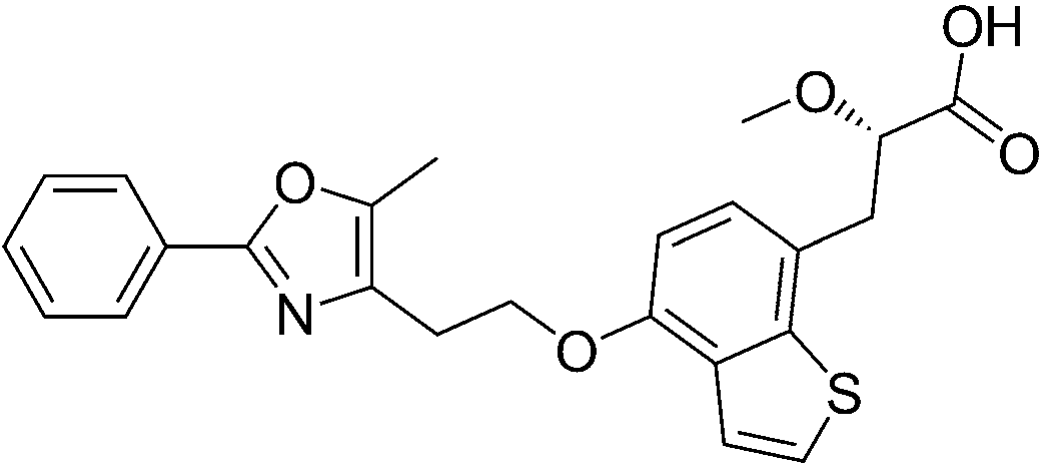	α/γ
Tesaglitazar	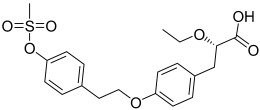	α/γ
Muraglitazar	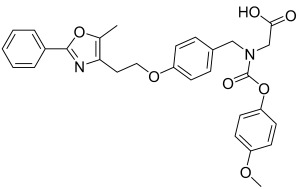	α/γ
Edaglitazone	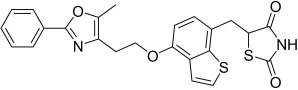	γ
Farglitazar	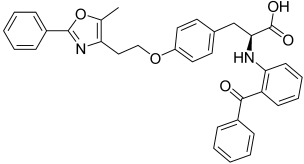	γ
Pioglitazone	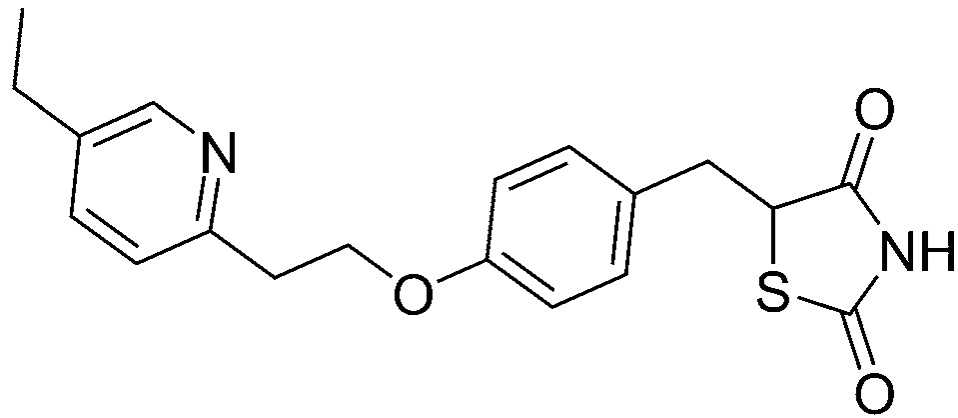	γ
Rosiglitazone	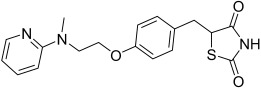	γ
RO4899100	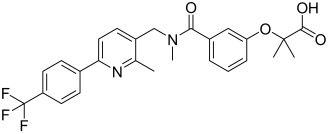	α
Fenofibric acid	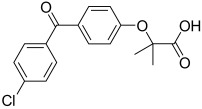	α
GW501516	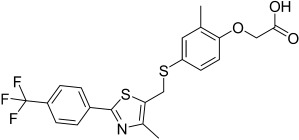	δ

## Results

### Effects on PPAR transcriptional activity

**Figure 2:**
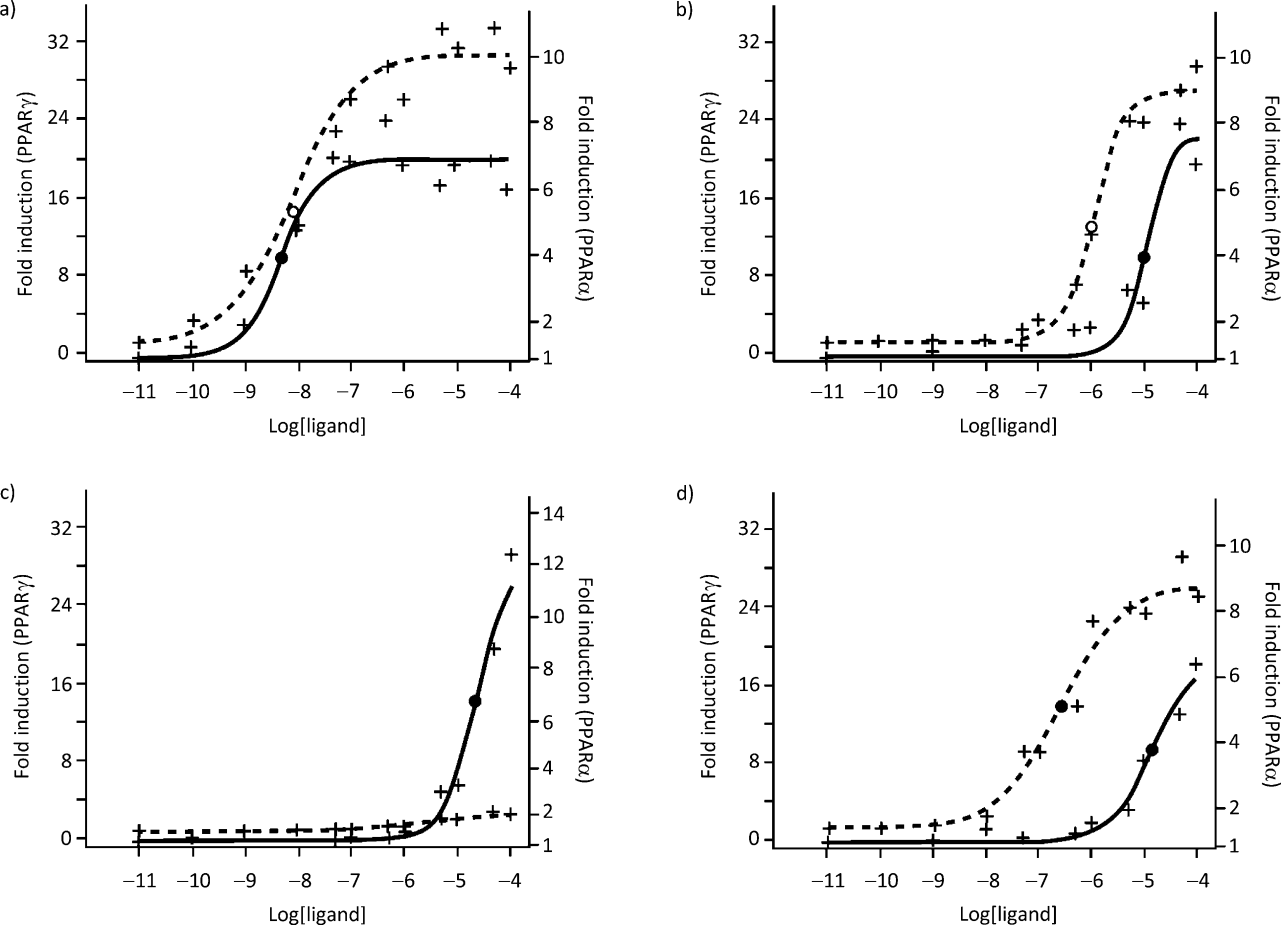
Transactivation profiles of a) aleglitazar, b) pioglitazone, c) fenofibric acid and d) rosiglitazone. Activation curve for PPARα (—); EC_50_ for PPARα (•); activation curve for PPARγ (- - - -); EC_50_ for PPARγ (○).

The results indicate that aleglitazar is a highly potent agonist of both PPARα and γ transcriptional activity, with half-maximal activation (EC_50_) values of 5 nm and 9 nm, respectively (Figure 2). The other dual PPARα/γ agonists tested, muraglitazar and tesaglitazar, are substantially less potent. EC_50_ values against PPARα and PPARγ were found to be 5680 nm and 243 nm for muraglitazar and 4780 nm and 3420 nm for tesaglitazar, respectively (Figure 2, Table 2), indicating selectivity towards PPARγ activation, particularly for muraglitazar.

**Table 2:** Summary of transcriptional activation potency and activity of different PPAR ligands.

Compd	PPARα	PPARγ	PPARδ
	EC_50_ [nm]	MFA^[a]^	EC_50_ [nm]	MFA^[a]^	EC_50_ [nm]	MFA^[a]^
Aleglitazar	5	7	9	29	376	6
Tesaglitazar	4780	12	3420	40	51 000	10
Muraglitazar	5680	11	243	25	16 400	13
RO4899100	193	15	19 900^[b]^	10^[b]^	51 500	12
Fenofibric acid	22 400	12	1470^[b]^	3^[b]^	1060	2.5^[b]^
Pioglitazone	11 600	7	1160	26	9210	30
Rosiglitazone	15 000	6.5	245	26	8630	40
GW501516^[b]^	NA	NA	NA	NA	36	100

[a] Maximum fold activation (MFA). [b] Value is inaccurate due to low maximal activation. [c] GW501516 activity was determined for PPARδ only. NA, not available.

Aleglitazar was also more potent towards PPARα than fenofibric acid (EC_50_=22 400 nm) and reference compound RO4899100 (EC_50_=193 nm) but had lower maximal activity, suggesting a partial PPARα agonist profile. Comparisons between the tested dual PPARα/γ agonists showed that aleglitazar elicits a lower maximum PPARα activation of sevenfold (Table 2) versus muraglitazar and tesaglitazar at 11- and 12-fold baseline, respectively, consistent with this hypothesis.

PPARγ was activated by aleglitazar to a similar degree as by the PPARγ agonists pioglitazone and rosiglitazone, achieving a maximum activity of 26–29-fold baseline. However, aleglitazar was the most potent of the three, with an EC_50_ value of 9 nm; values for rosiglitazone and pioglitazone were 245 nm and 1160 nm, respectively.

Aleglitazar was found to have a low potential to activate PPARδ (Table 2). Its maximum activation was low compared with the positive control GW501516 (sixfold and 100-fold maximum activity, respectively). Pioglitazone and rosiglitazone were less potent against PPARδ but had five times more activity than aleglitazar, with a maximum activation of >30-fold.

The EC_50_ values of aleglitazar towards both PPARα and γ observed in this study are lower than we previously reported; however, they are confirmed by time-resolved fluorescence resonance energy transfer (TR-FRET) data described in this report.^19^ The data reported here represent a more accurate analysis, since a more comprehensive 12-concentration (vs 8) dose–response analysis, including lower concentrations of ligands, was carried out.

### Cofactor recruitment

**Figure 3:**
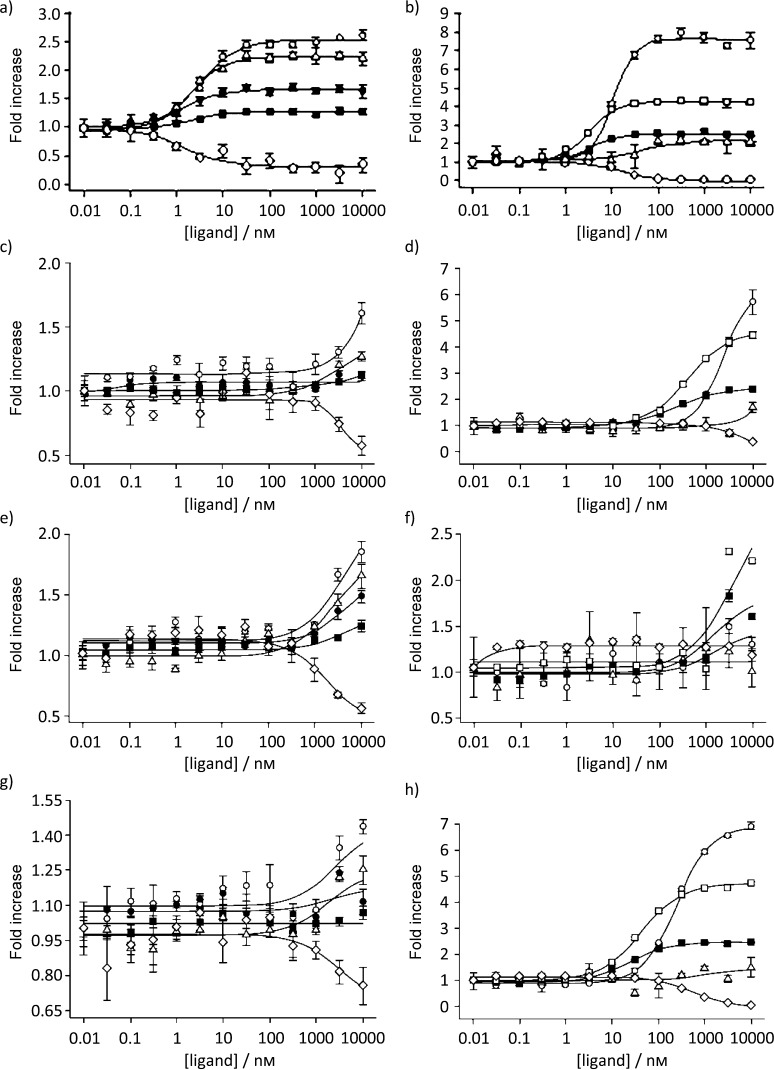
Cofactor recruitment profiles for assays of aleglitazar with a) PPARα and b) PPARγ; pioglitazone with c) PPARα and d) PPARγ; fenofibric acid with e) PPARα and f) PPARγ; rosiglitazone with g) PPARα and h) PPARγ. The cofactor peptides used in the assay were SRC1_M1 (○), SRC1_M4 (▪), TIF2_M1 (▴), TIF2_M2 (•), NcoR2 (⧫) and TRAP220_M2 (□). The *y*-axis denotes fold increase of TR-FRET signal versus baseline.

There were some differences in cofactor-peptide recruitment between PPARα and γ. Receptor-associated co-activator (RAC) 3_M1, nuclear receptor co-repressor (NCoR) 1, and silencing mediator of retinoid and thryoid receptor (SMRT) 1 were not recruited by either PPAR isoform (Table 3 and Figure 3). Eight of the 16 cofactor peptides tested were recruited to PPARα; these were steroid receptor co-activator (SRC) 1_M1, SRC1_M3, transcription intermediary factor (TIF) 2_M1, TIF2_M2, TIF2_M3, RAC3_M3, NCoR2, and SMRT2 (Table 3). The following peptides were not recruited by PPARα in the assay: SRC1_M2, SRC1_M4, RAC3_M2, cAMP responsive element binding protein (CREB)-binding protein (CBP) and thyroid hormone receptor-associated protein complex 220 kDa component (TRAP220). Cofactor peptides recruited to PPARγ were SRC1_M1, SRC1_M2, SRC1_M3, SRC1_M4, TIF2_M1, RAC3_M2, RAC3_M3, CBP, TRAP220, NCoR2 and SMRT2 (Table 3).

**Table 3:** Cofactor peptides used in the recruitment assay.

Cofactor^[a]^	Motif^[b]^	Cofactor peptides sequence^[c]^	Recruited by:
			PPARα	PPARγ
SRC1	M1	^623^DSKYSQTSHKLVQLLTTTAEQQLRH^647^	+	+
	M2^*^	^676^CPSSHSSLTERHKILHRLLQEGSPS^700^	−	+
	M3	^735^LDASKKKESKDHQLLRYLLDKDEKD^759^	+	+
	M4^*^	^1421^TSGPQTPQAQQKSLLQQLLTE^1441^	−	+
TIF2	M1	^630^SRLHDSKGQTKLLQLLTTKSD^650^	+	+
	M2^*^	^677^STHGTSLKEKHKILHRLLQDS^697^	+	−
	M3^*^	^736^SPKKKENALLRYLLDKDDTK^755^	+	−
RAC3	M1	^615^SKGHKKLLQLLTCSSD^630^	−	−
	M2^*^	^670^SNMHGSLLQEKHRILHKLLQNGNSP^694^	−	+
	M3	^730^PKKENNALLRYLLDRDDPSDV^750^	+	+
TRAP220	M2^*^	^637^GNTKNHPMLMNLLKDNPAQDF^657^	−	+
CBP	–^*^	^55^SGNLVPDAASKHKQLSELLRGGSGS^79^	−	+
NCoR	ID1	^2064^HRLITLADHICQIITQDFARNQVSS^2081^	−	−
	ID2	^2268^ADPASNLGLEDIIRKALMGSF^2289^	+	+
SMRT	ID1	^2129^HQRVVTLAQHISEVITQDYTRHHP^2152^	−	−
	ID2	^2331^AVQEHASTNMGLEAIIRKALMGKYD^2355^	+	+

[a] Steroid receptor co-activator 1 (SRC1); transcriptional intermediary factor 2 (TIF2); receptor-associated co-activator 3 (RAC3); thyroid hormone receptor-associated protein complex 220 kDa component (TRAP220); cAMP responsive element binding protein (CREB)-binding protein (CBP); nuclear receptor co-repressor (NCoR); silencing mediator of retinoid and thyroid hormone receptors (SMRT). [b] Peptides differentially recruited by only one of the PPARs are indicated by ✶. All other peptides were recruited by both PPARs (or neither, for example, RAC3_M1 peptide). [c] Peptides and corresponding amino acid position within each cofactor.

**Figure 4:**
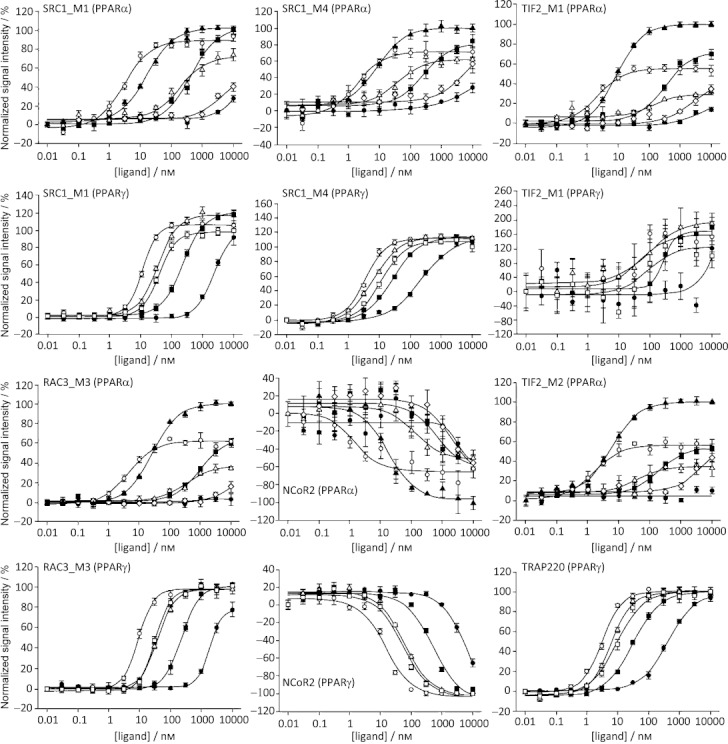
Selected dose–response profiles of ligands in PPARα and PPARγ assays with the given cofactor peptides. PPAR ligands used in the assays are aleglitazar (○), tesaglitazar (▪), muraglitazar (▵), pioglitazone (•), fenofibric acid (⧫), RO4899100 (▴) and edaglitazone (□). The *y*-axis shows signal intensity versus baseline, normalized to reference compounds RO4899100 (PPARα) or edaglitazone (PPARγ).

In cofactor binding assays, aleglitazar induced recruitment of peptides from cofactors SRC1, TIF2, RAC3, CBP and TRAP220, as well as displacement of NCoR2 and SMRT2 peptides. As expected, the dual PPARα/γ agonists were all able to recruit cofactors in assays using either PPARα or γ. While the efficacy (percentage maximal response compared with reference compounds) was broadly similar between aleglitazar and other dual PPARα/γ agonists (Table 4), aleglitazar was the most potent ligand (Figure 4).

**Table 4:** Cofactor-dependent recruitment potency and activity towards PPARα and PPARγ.

Compd	EC_50_ [nm] (delta ratio)^[a]^
	SRC1_M1	SRC1_M2	SRC1_M3	SRC1_M4	TIF2_M1	TIF2_M2	TIF2_M3	RAC3_M2	RAC3_M3	CBP	TRAP220	NCoR2	SMRT2
***PPARα***													
Aleglitazar	2.9 (94)	–	2.4 (53)	–	2.1 (53)	1.6 (53)	2.3 (56)	–	4.4 (62)	–	–	1.7 (−61)	14.1 (−51)
Tesaglitazar	427 (100)	–	111 (52)	–	326 (70)	182 (54)	377 (53)	–	941 (60)	–	–	646 (−54)	1780 (−26)
Muraglitazar	186 (74)	–	8.4 (23)	–	96.5 (31)	34 (33)	88.9 (29)	–	330 (35)	–	–	139 (−50)	563 (−28)
RO4899100^[b]^	16.1 (100)	–	4.9 (100)	–	8.6 (100)	5.9 (100)	12.7 (100)	–	25.9 (100)	–	–	18.5 (−100)	108 (−100)
Fenofibric acid	4363 (39)	–	3452 (47)	–	2161 (34)	5198 (44)	1293 (25)	–	>10 000 (16)	–	–	1903 (−55)	>10 000 (−2)
Edaglitazone	867 (41)	–	>10 000 (13)	–	945 (26)	294 (21)	3669 (32)	–	1917 (24)	–	–	1951 (−44)	790 (−20)
Pioglitazone	>10 000 (28)	–	>10 000 (13)	–	>10 000 (14)	>10 000 (10)	>10 000 (16)	–	>10 000 (3)	–	–	3217 (−54)	>10 000 (−10)
Rosiglitazone	2627 (20)	–	3070 (21)	–	>10 000 (13)	>10 000 (10)	>10 000 (14)	–	>10 000 (3)	–	–	3000 (−31)	>10 000 (−2)
***PPARγ***													
Aleglitazar	11.0 (105)	3.2 (95)	11.3 (99)	3.4 (109)	43.2 (154)	–	–	7.4 (90)	9.5 (102)	3.3 (105)	3.2 (97)	15.3 (−98)	14.4 (−99)
Tesaglitazar	226 (119)	24.4 (100)	196 (106)	25.5 (112)	274 (182)	–	–	110 (94)	233 (102)	13.9 (105)	31.6 (99)	537 (−96)	346 (−96)
Muraglitazar	35.6 (116)	5.7 (100)	37.4 (102)	6.1 (110)	94.3 (193)	–	–	16.5 (87)	39.5 (97)	4.3 (112)	6.2 (98)	63.4 (−98)	60.3 (−99)
RO4899100	>10 000 (16)	2079 (64)	>10 000 (12)	1451 (80)	>10 000 (25)	–	–	>10 000 (19)	>10 000 (17)	1994 (88)	4413 (57)	>10 000 (10)	>10 000 (−4)
Edaglitazone^[c]^	38.1 (100)	9.8 (100)	38.1 (100)	10.9 (100)	86.5 (100)	–	–	22.7 (100)	33.1 (100)	7.2 (100)	9.5 (100)	57.1 (−100)	41.6 (−99)
Farglitazar	8.6 (195)	3.8 (105)	12.1 (160)	3.3 (120)	16.1 (517)	–	–	6.5 (132)	8.7 (157)	3.0 (107)	3.5 (99)	13.6 (−97)	11.6 (−100)
Pioglitazone	2256 (92)	196 (93)	2115 (77)	222 (105)	>10 000 (122)	–	–	1071 (86)	1865 (77)	79.1 (101)	431 (94)	9422 (−66)	3701 (−63)
Rosiglitazone	240 (116)	18.5 (91)	241 (107)	23.5 (111)	951 (87)	–	–	125 (94)	271 (64)	10.4 (107)	41.3 (102)	497 (−101)	333 (−96)

[a] Value in parentheses is the delta ratio in % of delta ratio at a reference compound concentration of 10 μm. [b] For PPARα, RO4899100 was used as the reference compound. [c] For PPARγ, edaglitazone was used as the reference compound.

A different potency pattern resulted for aleglitazar with respect to recruitment of different cofactor peptides to PPARα versus γ. For example, aleglitazar showed a 3.8-fold greater potency in SRC1_M1/PPARα versus SRC1_M1/PPARγ recruitment, but a 20-fold greater potency in TIF2_M1/PPARα versus TIF2_M1/PPARγ recruitment (Table 4). This contrasts with the profile of both tesaglitazar and muraglitazar—tesaglitazar had a similar potency for cofactor complexes with both PPARα and γ, whereas muraglitazar showed a fivefold greater potency towards SRC1_M1/PPARγ compared with SRC1_M1/PPARα but a similar potency towards TIF2_M1/PPARα and TIF2_M1/PPARγ.

The ratio of PPARγ to PPARα EC_50_ values calculated from the cofactor recruitment assays indicates that aleglitazar exhibited a slightly greater potency towards PPARα, whereas both tesaglitazar and muraglitazar showed greater potency towards PPARγ (Table 5). This was in general agreement with the ratios of EC_50_ values as determined in the cell-based transcriptional transactivation assays (Table 2).

**Table 5:** Summary of cofactor recruitment potency of different PPAR ligands.

Compd	EC_50_^[a]^ [nm]	Ratio^[b]^
	PPARα	PPARγ	γ/α
Aleglitazar	2.0	10.3	5.1
Tesaglitazar	377	230	0.61
Muraglitazar	118	38	0.32
RO4899100	12.3	>10 000	>810
Fenofibric acid	4557	>10 000	>2.2
Edaglitazone	1053	35.6	0.034
Farglitazar	479	8.7	0.018
Pioglitazone	>10 000	2061	<0.21
Rosiglitazone	>10 000	256	<0.026

[a] Median EC_50_ values were determined from the EC_50_ value of each ligand against PPARα or PPARγ and select cofactors. For PPARα, these were SRC1_M1, SRC1_M4, TIF2_M1, TIF2_M2, RAC3_M3 and NCoR2. For PPARγ, the cofactors were SRC1_M1, SRC1_M4, TIF2_M1, RAC3_M3, TRAP220 and NCoR2. [b] Ratio of EC_50_ values for PPARγ/PPARα.

Cofactor recruitment studies showed that RO4899100 and fenofibric acid have a clear PPARα agonist profile, with exclusively PPARα activity and no PPARγ activity (Table 5). Aleglitazar was the most potent PPARα ligand compared with all compounds, including RO4899100 and fenofibric acid, irrespective of the cofactor employed. Interestingly, the maximal efficacy of aleglitazar in recruiting cofactor peptides was 53–62 % that of RO4899100, except for SRC1_M1, which was recruited by aleglitazar to a similar extent (94 %) as it was by RO4899100 (Table 4).

These data, along with the transactivation data, suggest that aleglitazar might be a partial PPARα agonist in comparison with RO4899100. To test this hypothesis, we performed recruitment/competition assays with TIF2_M2 and NCoR2 as representative co-activator and co-repressor peptides, respectively. The results confirmed that aleglitazar induces partial recruitment of TIF2_M2 versus full recruitment by RO4899100. Moreover, increasing concentrations of aleglitazar were able to compete with the full recruitment induced by RO4899100 at 100 nm down to the partial recruitment profile characteristic of aleglitazar alone (figure S1 a in the Supporting Information). Both aleglitazar and RO4899100 were able to completely displace NCoR2, demonstrating that both compounds fully occupy the PPARα receptors (figure S1 b in the Supporting Information). These results suggest that the differential recruitment of TIF2_M2 is indeed due to different conformations of the complexes of aleglitazar and RO4899100 with PPARα.

Rosiglitazone, pioglitazone, edaglitazone and farglitazar have a clear PPARγ agonist profile, with predominant PPARγ activity and little (edaglitazone/farglitazar) or no (rosiglitazone/pioglitazone) PPARα activity (Table 4). Furthermore, in assays with PPARγ, aleglitazar was a more potent agonist than the reference compounds edaglitazone, tesaglitazar or muraglitazar, and it exhibited superior potency to pioglitazone (Figure 4 b,d and Table 4). No significant differences in maximal efficacy were observed between the agonists, with the exception of farglitazar, the most efficacious ligand in the cofactor assays (Table 4).

The potency of several ligands (e.g., pioglitazone and rosiglitazone) differs when compared with earlier reports,^39^ but the activities are roughly in line with subsequent literature.^40^ Our study, employing two independent assays (cell-based transactivation and non-cell-based FRET), produced reasonably concordant data. For example, the EC_50_ value of rosiglitazone against PPARγ is 245 nm in the transactivation assay (Table 2) and 256 nm in the cofactor experiments (Table 5). The corresponding values for pioglitazone were 1160 nm and 2061 nm, respectively, while aleglitazar showed the highest potency at 9 nm and 10 nm, respectively. Notable disparities, for example, are values for muraglitazar and tesaglitazar, which showed higher potency in the cofactor recruitment assay compared with values determined from the results of transactivation experiments. These observations highlight the value of head-to-head comparative testing since comparisons across studies are complicated by alternative protocols, employing, for example, different cell backgrounds or full-length versus chimeric receptors.

## Discussion

The current study demonstrates that aleglitazar shows a potent and balanced activity for both PPARα and γ, as analyzed by transcriptional transactivation and cofactor recruitment assays. In transactivation assays, aleglitazar was found to have EC_50_ values of 5 nm and 9 nm against PPARα and γ, respectively. Aleglitazar was the most potent agonist for PPARα and γ of a panel of seven ligands, which included compounds in clinical use, such as fenofibrate and pioglitazone, and also the prototype dual PPARα/γ agonists muraglitazar and tesaglitazar.

The high and balanced potency of aleglitazar for both PPARα and γ can be rationalized from the X-ray co-crystal structures. The negatively charged carboxylate head group of aleglitazar is involved in four strong hydrogen bonds with PPARα and PPARγ, effectively minimizing desolvation penalties. While the TZD head group, as present in pioglitazone and rosiglitazone, is able to interact efficiently with PPARγ, it shows significantly reduced affinity for PPARα due to an amino acid difference (His 323 to Tyr 314) in this polar recognition region. The bulkier Tyr 314 present in PPARα results in a smaller ligand binding pocket, which can be much better accommodated by the smaller carboxylate head group of aleglitazar compared with the TZD head group. The remaining residues lining the extended ligand pocket are mostly hydrophobic. In comparison with pioglitazone and rosiglitazone, aleglitazar contains greater buried surface area, making additional hydrophobic interactions through an annulated thiophene at the central phenyl ring and an additional terminal phenyl moiety. Together, these features appear to explain the remarkably high and, most importantly, balanced affinity of aleglitazar for both PPARα and PPARγ.

The mechanisms by which PPARs regulate gene transcription are multifaceted. Current evidence suggests that binding of ligands to PPARs induces conformational changes that result in the release of co-repressors and the binding of co-activators, inducing the transcription of target genes. Numerous studies in rat and human primary hepatocytes and human HepG2 cells have documented differential but overlapping patterns of gene expression for different PPAR ligands.^35^–^37^ Similarly, aleglitazar induces transcriptional signatures different from those of other dual PPARα/γ treatments, for example with respect to expression of genes in lipid metabolism or stress pathways.^41^ Such data have led to suggestions that each ligand–receptor complex adopts a slightly different three-dimensional structure that results in a distinct pattern of cofactor recruitment and gene signature profile unique to each ligand.^32^, ^42^ Thus, pharmacologic differences between PPAR ligands could be due in part to differences in recruited cofactors (identity and/or potency). In this context, PPARγ co-activators have been described as being “adverse” or “beneficial”, depending on their pro-adipogenic or insulin-sensitizing effects.^2^ Co-activators that have been associated with possible adverse effects include TRAP220 and TIF2,^42^–^44^ and their recruitment has been suggested to lead to adipogenesis and/or insulin resistance. In contrast, co-activators such as SRC1 have been associated with beneficial effects such as increasing energy expenditure through thermogenesis.^44^

In the current study, the cofactor recruitment signature of aleglitazar with PPARα was similar to that observed with the reference compound RO4899100, except that aleglitazar is more potent, although with less efficacy (53–62 %). Cofactor recruitment/competition studies with aleglitazar versus RO4899100 confirmed this profile; thus, aleglitazar can be considered to be a potent partial PPARα agonist, especially when compared with RO4899100 and other dual-PPARα/γ agonists.

Qualitatively, the cofactor recruitment signature of aleglitazar on PPARγ was roughly in line with that of the other full PPARγ agonists (rosiglitazone and pioglitazone) and previously described dual PPARα/γ agonists (muraglitazar and tesaglitazar). Aleglitazar was able to recruit peptides derived from SRC1, TIF2, TRAP220 and several others with higher potency and to a similar extent to PPARγ. Thus, these results classify aleglitazar as a full PPARγ agonist as assessed by these methods. Indeed, the dose-dependent effects of aleglitazar on weight gain versus placebo observed in SYNCHRONY are in line with its ability to recruit TRAP220 and TIF2. Extrapolation of the current in vitro results to the preclinical and clinical scenario is challenging. One speculative possibility is that the higher potency of aleglitazar in recruiting SRC1 compared with TIF2 and TRAP220 peptides might partly explain the observation made in SYNCHRONY^24^ that treatment with aleglitazar (150 μg) resulted in a numerically lower degree of weight gain compared with 45 mg pioglitazone, although the study was not designed to assess significant differences between the two. However, an argument against this explanation is the observation that both pioglitazone and rosiglitazone also preferentially recruit SRC1 versus TIF2 peptides despite substantially lower overall potency. It could be that the partial agonist profile of aleglitazar on PPARα, combined with its ability to act as a balanced activator of PPARα/γ, underlies its favorable clinical profile observed in SYNCHRONY.

The results from the cofactor recruitment assay highlight distinct pharmacologic features of aleglitazar versus other PPARα/γ agonists and might explain, at least in part, the unique gene expression profile observed with the different ligands. However, it must be acknowledged that these data are derived using the ligand binding domains of the PPARs and LxxLL-containing peptide motifs of the cofactor panel. Cofactors such as SRC1 and TIF2 contain several binding motifs available for interacting with PPAR, and in full-length proteins with a complete cofactor–receptor interaction, these motifs might produce a synergistic effect not observed with the use of short, synthetic peptides.^45^, ^46^ Therefore, additional studies are required to confirm the effects using full-length cofactor proteins, including the finding that aleglitazar acts as a partial PPARα agonist.

It is well established that treatment of type 2 diabetes mellitus patients with TZD PPARγ agonists is associated with weight gain, in part due to increased adipogenesis/fat mass. Considerable research has focused on identifying PPARγ activators that maintain the beneficial metabolic features without increasing adipogenesis. One approach is the use of moderate activation of PPARγ, for example by differential co-activator recruitment with decreased adipogenic capacity,^42^ or via partial agonism of the receptor. The latter so-called selective PPAR modulation might alleviate the weight-gain effect, and possibly other side effects, associated with TZDs.^17^, ^42^, ^47^

Several selective PPAR modulators (MK0533,^48^ ATx008-001/FK614,^33^ MBX-102^33^ and INT131^32^) have progressed into clinical development. In particular, preclinical studies in animal models of diabetes showed INT131 to be efficacious in reducing plasma glucose while resulting in fewer side effects as compared with rosiglitazone.^32^, ^34^ However, a phase I multiple ascending-dose study showed that INT131 at the highest dose—10 mg per day for four weeks—caused significant weight gain as well as modest edema in 25 % of patients, concurrent with significant lowering of plasma glucose.^49^ Thus, the concept of using selective PPAR modulators to reduce side effects is intriguing, but the incidence of side effects in clinical trials for INT131, despite having an apparently improved safety profile in animal studies, demonstrates the complexity of mechanisms involved in PPAR cofactor recruitment and regulation of gene expression.

Recently, the role of classical receptor agonism in the action of PPARγ ligands has come under scrutiny as a cyclin-dependent kinase 5 (Cdk5)-mediated decrease in PPARγ phosphorylation was found to correlate with the antidiabetic effect of some PPARγ ligands (MRL24 and rosiglitazone) independently of receptor agonism.^50^ As no differences were observed in the DNA binding for different phosphorylation states of PPARγ, it was suggested that other factors, such as cofactor recruitment, might be regulated in a phosphorylation-dependent manner.^50^ It would be interesting to determine the effect of aleglitazar, and other ligands that show differential effects on adipogenesis/weight gain, on Cdk5-dependent phosphorylation sites on PPARγ and to analyze their correlation with cofactor recruitment. However, it must be noted that the investigations of changes in phosphorylation status have been limited to mainly preclinical studies, with assessment in only a small cohort of subjects treated with rosiglitazone. The same mechanism does not apply to either PPARα or PPARδ, suggesting transcriptional agonism will still be required for the additive, added lipid benefits of dual activation of PPARα along with PPARγ.

## Conclusions

The results of this study have characterized aleglitazar as a potent dual PPARα/γ agonist with a unique profile in terms of potency and balance, as evaluated in transactivation and cofactor recruitment assays. Furthermore, the in vitro binding and cofactor recruitment profile of aleglitazar show qualitative differences compared with the profiles of other agonists that might explain the effects of aleglitazar observed in preclinical and clinical studies.

## Experimental Section

**General**: The structures of aleglitazar, tesaglitazar, muraglitazar, 2-methyl-2-(3-{methyl-[2-methyl-6-(4-trifluoromethyl-phenyl)pyridin-3-ylmethyl]carbamoyl}phenoxy)propionic acid (RO4899100, an experimental PPARα-selective compound),^51^ fenofibric acid, edaglitazone, farglitazar, pioglitazone, rosiglitazone and GW501516 are shown in Table 1. RO4899100 and edaglitazone were selected as reference compounds for assays with PPARα and PPARγ, respectively. GW501516 was chosen as reference for PPARδ experiments. Compound stock solutions were prepared in dimethyl sulfoxide (DMSO) at a final concentration of 10 mm, such that the final concentration of DMSO in assays did not exceed 0.1 % *v*/*v*. LANCE Eu-W1024-labeled anti-glutathione-S-transferase (GST) antibody (3.9 μm) and SureLight allophycocyanine streptavidin were obtained from PerkinElmer (Waltham, MA, USA). Purified GST–PPARα and γ fusion proteins were produced in-house.

### Transactivation assay

*Expression plasmids*: The preparation of plasmid constructs expressing PPARα, γ and δ has been described previously.^52^ Briefly, the DNA binding domain of the yeast Gal4 transcription factor was fused in-frame to the N terminus of the ligand binding domains of human PPARα (amino acids 167–469), mouse PPARγ (amino acids 174–476) or human PPARδ (amino acids 139–442) receptors. Mouse and human PPARγ are 97 % identical in their ligand binding domains and 100 % identical in their ligand binding pockets.

*Assay protocol*: Baby hamster kidney cells (BHK21) were grown in Dulbecco’s modified Eagle’s medium (DMEM) containing 10 % fetal bovine serum (FBS) at 37 °C and 5 % CO_2_. Cells were distributed in six-well plates at a density of 10^5^ cells per well then transfected using FuGENE 6 reagent (Roche Molecular Biochemicals, Rotkreuz, Switzerland) with the pFR-Luc luciferase reporter plasmid and expression plasmids for PPARα, γ or δ ligand binding domains. Cells were harvested by trypsinization 6 h post-transfection and then distributed in 96-well plates at a density of 10^4^ cells per well. After incubation for 24 h to allow attachment of cells, the medium was removed and replaced with 100 μL of phenol red-free medium containing the test or reference compound. Following 24 h incubation in the presence of ligand, 50 μL of the medium was replaced with 50 μL of luciferase constant-light reagent (Roche Molecular Biochemicals) to lyse cells and initiate the luciferase reaction. Luminescence was detected in a TopCount microplate reader (PerkinElmer).

*Data analysis*: Comprehensive 12-concentration dose–response curves with quadruplicate values per concentration were produced for all compounds to ensure accurate comparative values. Transcriptional activation in the presence of a test compound was expressed as fold-change in luminescence compared with cells incubated at low, non-activating concentrations of the test compound. Data were calculated as the mean ± standard error of the mean (SEM) of luciferase values and were then converted to fold activation. Potencies for EC_50_ values of receptor transcriptional activity were calculated using the XLfit program (ID Business Solutions Ltd., Guildford, UK) using a one-site dose–response model.

### Time-resolved fluorescence resonance energy transfer assay

Homogeneous TR-FRET represents a highly sensitive and robust assay format for determination of peptide–peptide or peptide–protein interactions. TR-FRET assays were used here to determine the interaction signatures of the cofactor peptides (co-activators and co-repressors) with the purified ligand binding domains of PPARα and γ in response to all ligands.

*Cofactor peptides*: A total of 16 FRET peptides corresponding to potential PPAR interaction motifs previously identified in several transcriptional co-activators (SRC1, TIF2, RAC3, TRAP220 and CBP) and transcriptional co-repressors (NCoR and SMRT) were synthesized by Jerini (Berlin, Germany) or Biosynthan (Berlin, Germany). All cofactor-derived peptides were 20–25 amino acids in length (sequences given in Table 3) and were labeled with biotin separated by an aminohexanoic acid spacer at the N terminus. Stock solutions (200 μm in DMSO) were stored at −20 °C.

*Assay protocol*: Homogeneous TR-FRET was performed with either GST–PPARα (20 nm) or GST–PPARγ (20 nm) in each well in 384-well microtiter plates (Corning, Corning, NY, USA) by incubating for 60 min at 37 °C in the presence or absence of agonist and the individual cofactor (500 nm) and a cocktail containing Eu-W1024-labeled anti-GST antibody (0.76 nm) and allophycocyanine streptavidin (40 nm) in HEPES-buffered solution (50 mm HEPES (pH 7.4), 25 mm NaCl, 0.1 mg mL^−1^ fatty acid-free bovine serum albumin (BSA), 1 mm dithiothreitol). Fluorescence was quantified at an excitation wavelength of 337 nm and at emission intensities of 665 nm (allophycocyanine) and 620 nm (europium cryptate) using a NanoScan time-resolved fluorescence plate reader (IOM, Berlin, Germany).

### Ligand effects on cofactor peptide–PPAR interactions

In a pilot experiment, the binding ability of the 12 co-activator and four co-repressor peptides to PPARα and PPARγ was determined in the presence of 10 μm of each PPAR ligand (Table 3). Following identification of the subset of peptides that showed ligand-dependent recruitment to either PPARα or PPARγ, detailed concentration–response experiments were performed to calculate both the potency (EC_50_) and the maximal signal versus baseline for all peptides shown to be recruited to each PPAR in the pilot experiments.

*Data analysis*: Comprehensive 12-concentration dose–response curves with quadruplicate values per concentration were produced for all compounds on all PPARα– or PPARγ–cofactor pairs to ensure accurate comparative values. Data were calculated as the mean ± standard deviation (SD) for each concentration. Binding potency was determined as the EC_50_ value from the FRET signal for each ligand on each receptor–cofactor pair. Curves were fit to a one-site dose–response model using XLfit. Median EC_50_ values for each ligand were calculated from the mean EC_50_ values determined from several cofactor peptides. For PPARα, they were SRC1_M1, SRC1_M4, TIF2_M1, TIF2_M2, RAC3_M3 and NCoR2; for PPARγ, they were SRC1_M1, SRC1_M4, TIF2_M1, RAC3_M3, TRAP220 and NCoR2. For comparison of the magnitude of the TR-FRET signals induced by ligands as potentially indicative of specific ligand-induced conformations, the fold-change in signal for each PPAR–peptide combination versus baseline was calculated and normalized to the positive controls RO4899100 (PPARα) or edaglitazone (PPARγ), for which the values were set to 100 %.
