# Supplementary material for: Comparative Molecular Profiling of the PPARα/γ Activator Aleglitazar: PPAR Selectivity, Activity and Interaction with Cofactors
Source: ChemMedChem. 2012 Apr 4;7(6):1101–11. doi: 10.1002/cmdc.201100598 (PMC3504387; doi:10.1002/cmdc.201100598)
Supplement: Supplementary file 1 [file cmdc0007-1101-SD1.pdf]

CHEMMEDCHEM
Chemistry & Drug Discovery

Supporting Information

© Copyright Wiley-VCH Verlag GmbH & Co. KGaA, 69451 Weinheim, 2012

Comparative Molecular Profiling of the PPARα/γ Activator Aleglitazar: PPAR Selectivity, Activity and Interaction with Cofactors

Michel Dietz,[b] Peter Mohr,[c] Bernd Kuhn,[c] Hans Peter Maerki,[c] Peter Hartman,[d] Armin Ruf,[b] Jörg Benz,[b] Uwe Grether,[c] and Matthew B. Wright*[a]

cmdc_201100598_sm_miscellaneous_information.pdf

Supplementary Figure 1: Aleglitazar is a partial PPAR-α agonist compared with RO4899100 in cofactor activation assays. Dose–response profiles of ligand competition studies with PPAR-α and a) TIF2_M1 and b) NCoR. Ligands used in the assay were RO4899100 (triangles), aleglitazar (open circles), RO4899100 co-incubated with 100 nM RO4899100 (open squares) and aleglitazar co-incubated with 100 nM RO4899100 (filled squares).

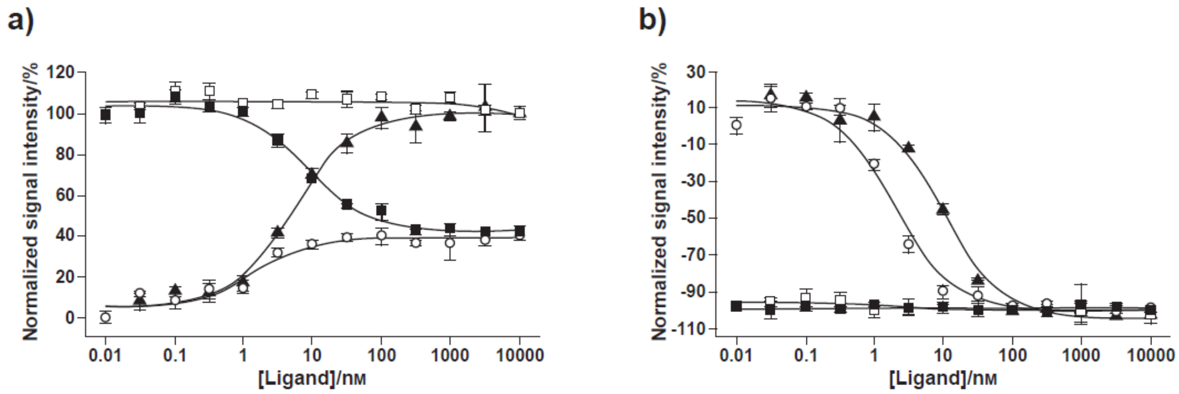

The TR-FRET assays were conducted as described in the Methods: briefly, to a solution of GST-PPAR-α (20 nM), aleglitazar or RO4899100 at the specified concentrations was added, followed by RO4899100 (100 nM). This was followed by addition of cofactors TIF2_M1 or NCoR2 (500 nM) and finally the Eu-anti-GST/SA-APC mix (0.76 nM/40 nM). Subsequent incubation and measurement of fluorescence signals were conducted as described in the Methods.
